# Effects of cyclosporine A pretreatment of deceased organ donors on kidney graft function (Cis-A-rein): study protocol for a randomized controlled trial

**DOI:** 10.1186/s13063-018-2597-4

**Published:** 2018-04-17

**Authors:** Jean-Christophe Orban, Eric Fontaine, Elisabeth Cassuto, Karine Baumstarck, Marc Leone, Jean-Michel Constantin, Carole Ichai, for the AzuRéa network

**Affiliations:** 1 Université Côte d’Azur, Service de Réanimation Polyvalente, Hôpital Pasteur 2, Nice, France; 2 Unité de Nutrition artificielle, CHU de Grenoble, Grenoble, France; 3 Transplantation Rénale, Hôpital Pasteur 2, CHU Nice, Nice, France; 4 Délégation à la Recherche Clinique et à l’Innovation, Assistance Publique Hôpitaux de Marseille, Marseille, France; 5 Service Anesthésie et Réanimation, Hôpital Nord, Aix Marseille Université, Assistance Publique Hôpitaux de Marseille, Marseille, France; 6 Département de Médecine Péri-opératoire, CHU de Clermont-Ferrand, Clermont-Ferrand, France

**Keywords:** Cyclosporine A, Preconditioning, Transplantation, Kidney, Delayed graft function

## Abstract

**Background:**

Renal transplantation represents the treatment of choice of end-stage kidney disease. Delayed graft function (DGF) remains the most frequent complication after this procedure, reaching more than 30%. Its prevention is essential as it impedes early- and long-term prognosis of transplantation. Numerous pharmacological interventions aiming to prevent ischemia-reperfusion injuries failed to reduce the rate of DGF. We hypothesize that cyclosporine as an early preconditioning procedure in donors would be associated with decreased DGF.

**Methods:**

The Cis-A-rein study is an investigator-initiated, prospective, multicenter, double-blind, randomized, controlled study performed to assess the effects of a donor preconditioning with cyclosporine A on kidney grafts function in transplanted patients. After randomization, a brain dead donor will receive 2.5 mg kg^−1^ of cyclosporine A or the same volume of 5% glucose solution. The primary objective is to compare the rate of DGF, defined as the need for at least one dialysis session within the 7 days following transplantation, between both groups. The secondary objectives include rate of slow graft function, mild and severe DGF, urine output and serum creatinine during the first week after transplantation, rate of primary graft dysfunction, renal function and mortality at 1 year. The sample size (*n* = 648) was determined to obtain 80% power to detect a 10% difference for rate of DGF at day 7 between the two groups (30% of the patients in the placebo group and 20% of the patients in the intervention group).

**Discussion:**

Delayed graft function is a major issue after renal transplantation, impeding long-term prognosis. Cyclosporine A pretreatment in deceased donors could improve the outcome of patients after renal transplantation.

**Trial registration:**

ClinicalTrials.gov, ID: NCT02907554 Registered on 20 September 2016.

**Electronic supplementary material:**

The online version of this article (10.1186/s13063-018-2597-4) contains supplementary material, which is available to authorized users.

## Background

Renal transplantation represents a major treatment for end-stage kidney disease patients. In France, the number of kidney transplantations increases to reach more than 3000 per year [1]. The most frequent complication of such a procedure is the delayed graft function/slow graft function (DGF/SGF). Delayed graft function is commonly defined as the need for at least one dialysis session within the first week after transplantation [2]. Delayed and slow graft functions account for 30% and 40–60% of renal transplantations, respectively, with variations depending on the presence of risk factors [2–4]. Alteration of the long-term renal function represents the main problem related to these complications [5]. The pathophysiology of DGF/SGF involves ischemia-reperfusion injuries [6]. During the transplantation procedure, the kidneys suffer from warm (harvesting and grafting before vascular anastomosis) and cold ischemia (preservation) followed by reperfusion. The mechanisms of renal damages are complex, multifactorial and interdependent leading to biochemical, structural and functional alterations. The association of hypoxic and ischemia-reperfusion injuries decreases renal blood flow, increases oxidative stress and triggers the release of pro-inflammatory mediators which activate leukocyte adhesion and immune phenomena. It seems that reperfusion induces sustained abnormalities leading to tissue damage characterized by apoptotic and necrotic cell death and finally renal dysfunction. To our knowledge, several clinical interventions aiming to decrease such damages have reported conflicting results on the incidence of DGF [7, 8].

Mitochondria play a central role in the pathophysiology of ischemia-reperfusion injuries. The homeostatic disturbances associated with this phenomenon lead to the formation of the permeability transition pore (PTP) and its subsequent opening between the internal and the external mitochondrial membranes. This has consequences for mitochondrial depolarization, energetic failure, oxidative stress and the release of proapoptotic factors. The composition of the PTP is debated and the only certainty is the presence of a regulatory protein, cyclophillin D. The inhibition of the binding of this protein to the PTP prevents the opening of this channel, and potentially the deleterious biochemical cascade.

Cyclosporine A is largely administered for immunosuppression that is mediated by the inhibition of calcineurin. This medication served for the development of solid organ transplantation, especially kidneys. Subsequent research unveiled other properties including the inhibition of the opening of the PTP by its binding to cyclophilin D. This property was used to decrease ischemia-reperfusion injuries in different organs and experimental models [9, 10]. In a rabbit model of cardiac arrest induced by asphyxia, cyclosporine A decreased the magnitude of cardiac, liver and renal dysfunction [11]. These results were associated with a reduction in the PTP opening and a restoration of oxidative phosphorylation. Cyclosporine A also improved the organ and the mitochondrial functions as well as histological injuries in models of liver and cardiac transplantation [12]. Three studies reported conflicting results concerning the potential benefits of cyclosporine A in rat kidney transplantation models [13–15]. This discrepancy could be explained by a prolonged cold ischemia time (24 h) in the negative study [15]. Based on these data, we hypothesize that a cyclosporine A preconditioning in brain-dead donors, by inhibiting the PTP opening, will improve the outcome of patients undergoing renal transplantation. The primary objective of the study is to evaluate the effects of such a preconditioning treatment administered in organ donors with a beating heart on the rate of DGF in the recipient, defined by the need of at least one dialysis session during the first week after transplantation. The main secondary objectives are the rate of SGF during the first week, urine output and serum creatinine during the first week, the rate of primary graft dysfunction, graft function and the mortality at 1 year after transplantation.

## Methods/design

### Trial design

**Fig. 1:**
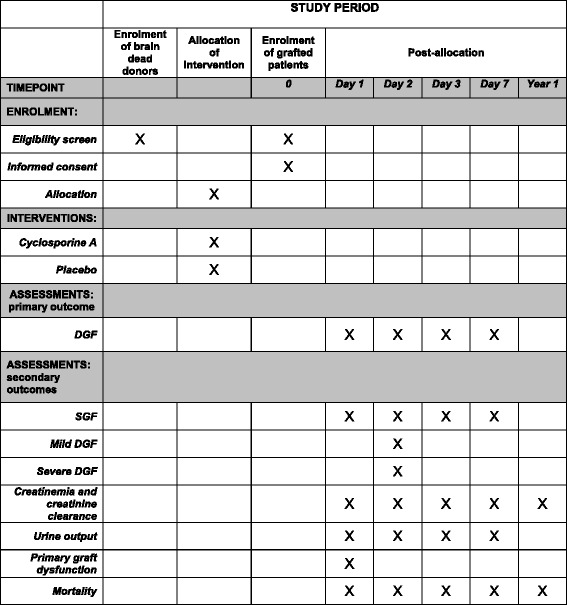
Overview of enrollment, interventions and assessments of the Cis-A-rein study. *DGF* delayed graft function, *SGF* slow graft function

“Cis-A-Rein” is an investigator-initiated, prospective, multicenter, double-blind, randomized controlled versus placebo study performed to assess the effects of a donor preconditioning with cyclosporine A on kidney grafts function in transplanted patients. After randomization, cyclosporine A or a placebo will be given to brain-dead patients with a beating heart before organ harvesting. The function of the kidneys procured from these patients will be analyzed in the recipients of this graft. The study protocol was designed using the recommendations of the Consolidated Standards of Reporting Trials (CONSORT) Statement. It is described as required by the SPIRIT guidelines to ensure consistent reporting of clinical trials (Additional file 1). Figure 1 reports the overview of the enrollment, interventions and assessments of the trial.

### Selection of participants

- ➢ Donor inclusion criteria
  - ○ Brain death with a beating heart
  - ○ Age between 18 and 70 years
- ➢ Donor exclusion criteria
  - ○ Contra-indications for organ harvesting (infections, cancer, etc.)
  - ○ Chronic kidney disease
  - ○ Refusal of organ donation by the donor (confirmed by the French National Register or reported by the next-of-kin)
  - ○ Inclusion in another protocol
- ➢ Recipient inclusion criteria
  - ○ Age between 18 and 70 years
  - ○ Indication of kidney transplantation
  - ○ Written informed consent given
- ➢ Recipient exclusion criteria
  - ○ Indication of double kidney transplantation
  - ○ Indication of multiorgan transplantation

### Randomization and blinding

Computer-generated randomized lists will be drawn up before the onset of the study using a permuted block design, under the responsibility of the Clinical Research Unit (Unité Aide Méthodologique à la Recherche Clinique, AP-HM, France). Randomization (1:1 allocation ratio) will be stratified by center (*n* = 16) and the number of kidneys per donor (two modalities, one or two). Randomization is centralized, web-based and accessible 24 h a day according to the allocation list. A randomization number will be allocated to each patient. The intensivist and the nurse in charge of the donor with a beating heart will be blinded regarding the allocation of treatment (preparation by an intensive care unit (ICU) nurse or a clinician not in charge of the donor). The statistician, the transplanted patients and the clinicians in charge of the transplanted patient will all be blinded concerning the treatment allocation.

### Trial intervention

After randomization, donors with a beating heart will receive a single dose of 2.5 mg kg^−1^ of cyclosporine A (diluted to obtain a 5 mg mL^−1^ concentration corresponding to an infusion of 0.5 mL kg^−1^; Sandimmun**®**, Novartis Pharma, Rueil-Malmaison, France) or an equivalent volume of placebo (glucose 5% solution 0.5 mL kg^−1^) in the 2 h before the harvesting of kidneys in the operating theater. The management of the donors, the organs and the transplanted patients will be performed according to national guidelines [16] similarly in both groups.

### Outcomes

- ➢ Primary outcome: rate of DGF defined by the need for at least one dialysis session within the first 7 days after kidney transplantation
- ➢ Secondary outcomes:
  - ○ Rate of SGF defined by a urine output of less than 1000 mL on day 1 after transplantation, or a decreased of less than 30 and 70% in creatinine plasma concentration, respectively, on days 3 and 7 after transplantation
  - ○ Combined rate of DFG + SGF
  - ○ Mild DGF defined by a serum creatinine reduction ratio from post-transplant day 1 to day 2 less than or equal to 30% plus 24-h urine creatinine excretion on day 2 greater than 1000 mg
  - ○ Severe DGF defined by a serum reatinine reduction ratio from post-transplant day 1 to day 2 less than or equal to 30% plus 24-h urine creatinine excretion on day 2 less than or equal to 1000 mg
  - ○ Duration of in-hospital length of stay (post-transplantation hospitalization)
  - ○ Daily urine output on days 1, 3 and 7
  - ○ Creatininemia and estimated creatinine clearance by the Modification of Diet in Renal Disease (MDRD) formula [17] on days 1, 3 and 7
  - ○ Rate of primary graft dysfunctions on day 1 defined by the absence of immediate renal function (anuria)
  - ○ Time between transplantation and normalization of graft function in patients presenting a DGF
  - ○ Function of kidney transplants at 1 year: kidney graft dysfunction defined by a clearance creatinine less than 30 mL min^−1^ estimated by the MDRD formula or total dysfunction defined by a persistent hemodialysis
  - ○ In-hospital and 1-year mortality rate of transplanted patients

### Pharmaceutical aspects

The clinical trial pharmacist of the promoting hospital will package all study drugs in trial packs. Standard operating procedures (SOPs) have been developed for every stage of a controlled drug’s journey from procurement (ordering, receipt and transport) to safe storage, supply, administration, destruction and for dealing with an incident. The SOPs will be accessible to the staff at all times.

### Statistical analysis

The methodological support will be provided by the Clinical Research Unit of Marseille University Hospital (Unité Aide Méthodologique à la Recherche Clinique, AP-HM, France).

#### Sample size

The sample size was determined to obtain 80% power to detect a 10% difference for rate of DGF (as defined in the primary endpoint section) at day 7 between the two groups, as this difference is considered to be clinically significant. In accordance with previous studies [7, 8], we hypothesized that 30% of the patients in the placebo group and 20% of the patients in the intervention group will develop DGF. With the threshold for statistical significance set at a *p* value of 0.05 (two-sided alpha), these calculations showed that 588 participants (recipients) are needed (294 per group). Assuming that a potential 10% of participants will be lost to follow-up (for the primary or the secondary endpoints), a total of 648 recipients needs to be included. Secondary outcomes will be compared between the two groups: using the chi^2^ test or Fisher’s exact test for categorical variables and Student’s *t* test for continuous variables.

#### Data analysis

The data will be analyzed using SPSS version 17.0 software. The patients found to be eligible but not included in the study will be described and compared with the included patients. The patients who present at least one of the following conditions will be not in the final analysis: patients inappropriately included despite not providing consent and patients who remove their consent. The full analysis population (including all subjects who will be randomized and will be at least evaluated at baseline) will be used in the primary analysis, and the per-protocol population (including all subjects who will be randomized and will not have major protocol deviations) will be used in the secondary analysis to assess the robustness of the results. No interim analysis is planned. The normality of these parameters will be estimated using frequency histograms and the Shapiro test. The baseline parameters will be described for the two groups (“control” and “intervention”).

The analysis of the primary endpoint will consider the proportion of DGF for each group. These proportions will be compared using the chi^2^ test or Fisher’s exact test for categorical variables (primary analysis). Multivariate analysis (secondary analysis) using logistic regression models will be performed to determine variables potentially linked to DGF (donor’s variables: age, norepinephrine use, diabetes, high blood pressure; procurement variables: cold and warm ischemia durations; recipient’s variables: weight, dialysis duration).

### Data registration

Data will be entered into the web-based electronic Case Report Form (eCRF) (RedCap, Vanderbilt University, Nashville, TN, USA) by trial or clinical personnel under the supervision of the trial site investigators at each participating center. The following data reported to influence the outcome of kidney transplantation will be registered at the different stages of the procedure:

- ➢ In donors with a beating heart:
  - ○ Demographic data (age, sex, Body Mass Index (BMI), ethnic group, cause of death)
  - ○ Significant medical history (chronic kidney disease, hypertension, diabetes, alcohol abuse)
  - ○ Detailed SOFA score in the last 24 h before organ harvesting
  - ○ Significant biochemical abnormalities: hypernatremia > 160 mmol L^−1^, coagulation abnormalities (disseminated intravascular coagulation)
  - ○ Renal function, assessed by blood urea nitrogen levels, creatininemia and 24-h creatinine clearance in the last 24 h prior to the harvesting
  - ○ Immunological and viral status: Epstein-Barr virus (EBV) serology, number of HLA incompatibility
- ➢ During kidney harvesting, preservation and transplantation
  - ○ Preservation data: type of preservation solution, use of a perfusion machine, cold ischemia time
  - ○ Intraoperative data: hemodynamic stability (systolic blood pressure < 80 mmHg, need for vasopressors), warm ischemia time, technical problems (duration of anastomose, surgical difficulties, hemorrhage)
- ➢ In transplanted patients
  - ○ Demographic data (age, sex, BMI, blood group, ethnic characteristic)
  - ○ Significant medical history: hypertension, cardiac failure, diabetes, cause of the kidney disease, previous transplantation(s), duration of dialysis before transplantation, duration on the transplantation waiting list
  - ○ Viral (C hepatitis) and immunological (anti-donor antibodies) status
  - ○ Dialysis during the first week after transplantation
  - ○ Creatininemia and estimated creatinine clearance (MDRD) on days 1, 3 and 7 after transplantation
  - ○ 24-h urine output on days 1, 3 and 7 after transplantation
  - ○ Recipient and graft survival at 1 year

### Data handling and retention

Data will be handled according to French law. All original records (including consent forms, reports of suspected unexpected serious adverse events, and relevant correspondences) will be archived at the trial sites for 15 years. The trial database will be anonymized and maintained for 15 years.

### Enrollment and timeline

2015 to 2017: protocol, approvals from the ethics committee and trial tool development (eCRF, randomization system).

2017 to 2019: inclusion of patients from 16 French University Hospitals.

2019: cleaning and closure of the database.

Early 2020: data analyses, writing of the manuscript and submission for publication.

### Publication plan

The trial is registered at www.Clinicaltrials.gov. After trial completion, the manuscript will be submitted for publication. The listing of authors will be as follows: JC Orban, first author; E Fontaine, second author; E Casutto, third author; K Baumstarck, fourth author; M Leone, fifth author; JM Constantin, penultimate author and C Ichai, last author. The inclusion of “for AzuRéa network investigators” will be added at the end of the author list. The responsibles of the five ICUs and the five nephrology units for having included the largest number of patients will be granted authorship. The other investigators will be listed in the collaborator list.

## Discussion

Transplantation represents the best option to improve the prognosis and the quality of life of end-stage renal disease patients. Delayed graft function may complicate this procedure and thus worsens significantly the long-term prognosis. Numerous interventions failed to decrease the rate of this complication [8, 18]. This trial investigates the potential benefits of a pharmacological preconditioning procured by cyclosporine A. The dose of this molecule selected in our study (2.5 mg kg^−1^) is lower than that used in immunosuppressive protocols. Actually, the inhibition of PTP opening is achieved with low doses and does not involve an immunosuppressive effect. Moreover, previous clinical trials used this dosage without adverse effects, particularly on renal function [19]. The time of administration of cyclosporine is a critical question. Numerous studies have reported the deleterious effects of ischemia-reperfusion with the majority of lesions occurring during reperfusion. Thus, the best protective effects were obtained with different interventions before ischemia and/or reperfusion [9, 10]. In the transplantation model, this objective will be reached with the administration of cyclosporine A in the brain-dead donor before harvesting of the kidneys. In this case, cyclosporine A will be present at the site of effect before ischemia and reperfusion. Last, we selected the renal transplantation model because the kidney is the most frequently grafted organ. Moreover, the occurrence of DGF represents a significant complication impeding the short- and the long-term prognosis. An intervention decreasing this complication will improve the outcome of renal transplantation, which has major public health implications.

## Trial status

The study has been recruiting since 19 December 2017.

## Additional file

Additional file 1: SPIRIT 2013 Checklist: recommended items to address in a clinical trial protocol and related documents. (DOC 121 kb)

## Abbreviations

BMI: Body mass index
DGF: Delayed graft function
PTP: Permeability transition pore
SGF: Slow graft function
